# Different Dietary N-3 Polyunsaturated Fatty Acid Formulations Distinctively Modify Tissue Fatty Acid and N-Acylethanolamine Profiles

**DOI:** 10.3390/nu13020625

**Published:** 2021-02-15

**Authors:** Elisabetta Murru, Paula A. Lopes, Gianfranca Carta, Claudia Manca, Armita Abolghasemi, José L. Guil-Guerrero, José A. M. Prates, Sebastiano Banni

**Affiliations:** 1 Department of Biomedical Sciences, University of Cagliari, 09042 Monserrato, Cagliari, Italy; m.elisabetta.murru@gmail.com (E.M.); giancarta@unica.it (G.C.); claumanca@hotmail.com (C.M.); armita.abolghasemi@usherbrooke.ca (A.A.); 2 CIISA—Centro de Investigação Interdisciplinar em Sanidade Animal, Faculdade de Medicina Veterinária, Universidade de Lisboa, Avenida da Universidade Técnica, Pólo Universitário do Alto da Ajuda, 1300-477 Lisboa, Portugal; ampalopes@fmv.ulisboa.pt (P.A.L.); japrates@fmv.ulisboa.pt (J.A.M.P.); 3 Departamento de Tecnología de Alimentos, Universidad de Almería, 04120 Almería, Spain; jlguil@ual.es

**Keywords:** N-acylethanolamides (NAE), dietary n-3 PUFA, vegetable oils, marine oils, microalga oils

## Abstract

We investigated the influence of different dietary formulation of n-3 polyunsaturated fatty acids (PUFA) on rat tissue fatty acid (FA) incorporation and consequent modulation of their bioactive metabolite N-acylethanolamines (NAE). For 10 weeks, rats were fed diets with 12% of fat from milk + 4% soybean oil and 4% of oils with different n-3 PUFA species: soybean oil as control, linseed oil rich in α-linolenic (ALA), *Buglossoides arvensis* oil rich in ALA and stearidonic acid (SDA), fish oil rich in eicosapentaenoic acid (EPA) and docosahexaenoic acid (DHA), *Nannochloropsis* microalga oil rich in EPA or *Schizochytrium* microalga oil rich in DHA. FA and NAE profiles were determined in plasma, liver, brain and adipose tissues. Different dietary n-3 PUFA distinctively influenced tissue FA profiles and consequently NAE tissue concentrations. Interestingly, in visceral adipose tissue the levels of N-arachidonoylethanolamide (AEA) and N-docosahexaenoylethanolamide (DHEA), NAE derived from arachidonic acid (AA) and DHA, respectively, significantly correlated with NAE in plasma, and circulating DHEA levels were also correlated with those in liver and brain. Circulating NAE derived from stearic acid, stearoylethanolamide (SEA), palmitic acid and palmitoylethanolamide (PEA) correlated with their liver concentrations. Our data indicate that dietary n-3 PUFA are not all the same in terms of altering tissue FA and NAE concentrations. In addition, correlation analyses suggest that NAE levels in plasma may reflect their concentration in specific tissues. Given the receptor-mediated tissue specific metabolic role of each NAE, a personalized formulation of dietary n-3 PUFA might potentially produce tailored metabolic effects in different pathophysiological conditions.

## 1. Introduction

An optimal balance among dietary fatty acids (FA) is widely recommended, in particular the intake of n-6 and n-3 polyunsaturated fatty acids (PUFA) in a 4 to 1 ratio. The therapeutic and preventive benefits of dietary n-3 PUFA have been well documented and most evidence applies to n-3 highly unsaturated fatty acids (HUFA), such as eicosapentaenoic acid (EPA, 20:5n-3) and docosahexaenoic acid (DHA, 22:6n-3), occurring in marine-sourced products [1,2,3].

Because dietary preferences are resilient and it is difficult to recommend an increase in fish products intake to some consumer groups, a possible solution could be to increase n-3 PUFA intake through vegetable products rich in α-linolenic acid (ALA, 18:3n-3), the precursor of n-3 HUFA. However, today is evident that dietary n-3 PUFA possess, individually, distinct biological properties. Several studies have already demonstrated that the conversion of ALA into DHA is very limited (10–15%) because ALA at high concentrations saturates the Δ6-desaturase enzyme, implicated in the conversion of ALA into stearidonic acid (SDA, 18:4n-3) and 24:5n-3 to 24:6n-3 [4], which are metabolic precursors of DHA in both humans and rodents [5,6,7]. Therefore, EPA and DHA might be more efficiently incorporated into cell membranes through dietary intake [1,8].

In addition, ALA competes with linoleic acid (LA, 18:2n-6) through a series of desaturation and elongation steps, to form EPA and arachidonic acid (AA, 20:4n-6) respectively, causing a competition between n-3 and n-6 biosynthesis [2]. The tissue balance between the two families is therefore critical. In this regard, as an efficient means of increasing EPA concentrations in tissues, it has been suggested that dietary SDA, either from terrestrial plants or from *Buglossoides arvensis* seeds, was more efficiently converted to EPA than ALA [9,10,11,12,13]. However, other reports did not observe any increase of DHA levels as a result of SDA supplementation [12]. In a previous study, we showed that in rats both ALA- and SDA-rich hypercaloric diets increased tissue n-3 HUFA contents leading to a marked improvement on the n-6/n-3 ratio, although lacking effects on DHA biosynthesis [14]. These data were also confirmed in humans supplemented with 4 g/day of linseed oil for two months, in which the conversion to DHA was only 13% [15].

Based on the current knowledge on the occurrence of n-3 HUFA in microalgae, much attention has been directed for microalgae lipids as an alternative to fish oil supplements to improve n-3 HUFA levels in tissues [16,17,18]. However, it remains controversial whether algae n-3 HUFA constitute a better n-3 source than fish oil. In a previous study, we have investigated the influence of n-3 HUFA rich-diets (EPA from *Nannochloropsis* and DHA from *Schizochytrium* oils) in comparison with combined EPA- and DHA-rich fish oils on tissues FA profile of rat. We reported that a fish oil diet is more effective in causing tissue incorporation of EPA and DHA than microalgae oils from *Nannochloropsis* and *Schizochytrium*, respectively [19].

The physiological properties of n-3 HUFA have been attributed to several mechanisms linked to different metabolic pathways associated with obesity, including insulin resistance, liver and heart steatosis, and hypertension [20,21]. n-3 HUFA-derived NAE, such as *N*-eicosapentaenoylethanolamine (EPEA) from EPA and *N*-docosahexaenoylethanolamine (DHEA) from DHA have been shown anti-inflammatory and synaptogenic properties [22,23]. In addition, we also have shown that dietary n-3 HUFA can modify NAE levels in several tissues [24], in animal models [25,26], humans [27,28] and in vitro cell cultures [29]. In fact, dietary EPA and DHA, particularly in the form of phospholipids, by competing for their incorporation with AA, were able to decrease *N*-arachidonoylethanolamine or anandamide (AEA), the AA-derived NAE, which is responsible for some metabolic disorders. However, AEA is involved in different intracellular transduction pathways influencing numerous physiological functions, including neuronal development, neuromodulator processes, energy metabolism, and cardiovascular, respiratory and reproductive functions [30,31,32].

In rodents, dietary lipids having adequate n-6/n-3 PUFA ratios are currently believed as one means to control appetite and obesity, and to improve skeletal muscle response to glucose and insulin sensitivity by modifying endocannabinoid levels [30,31,32].

The primary purpose of this study was to investigate whether different dietary n-3 PUFA formulations from vegetable or marine oils can influence n-3 HUFA incorporation and modulate the biosynthesis of NAE in key metabolic tissues of Wistar rats. Moreover, we evaluated whether tissue concentrations of some NAE species are correlated to their precursors and also whether different NAE concentrations in plasma correlate with their own tissue concentrations.

## 2. Materials and Methods

### 2.1. Experimental Diets

The experimental diets were manufactured at CEIA3-Universidad de Almería (Service of Experimental Diets, http://www.ual.es/stecnicos_spe (accessed on 7 January 2021 )). Diets were based on AIN-93M standard formulation for rodents with modified lipid composition (approximately 20% of total fat, w/w), as follows: milk fat diet (MilkFat), a control with 12% of fat from milk and 8% from soybean oil; milk fat diet plus linseed oil (LSO) with 12% fat from milk, 4% from soybean oil and 4% from linseed oil rich in ALA; milk fat diet plus *Buglossoides arvensis* oil (Buglos) with 12% fat from milk, 4% from soybean oil and 4% from ALA- and SDA-rich Buglos; milk fat diet plus cod liver oil (FO) with 12% from milk fat, 4% from soybean oil and 4% from cod liver oil rich in EPA and DHA; milk fat diet plus *Nannochloropsis* microalga oil (Nanno) with 12.5% milk fat, 5.9% from soybean oil and 2.4% from EPA-rich Nanno oil; milk fat diet plus *Schizochytrium* microalga oil (Schy) with 12.2% from milk fat, 6.5% from soybean oil and 1.8% from DHA-rich Schy oil. Diet ingredients were purchased at Dyets inc. (Bethlehem, PA, USA). Buglossoides oil was obtained from *Buglossoides arvensis* seeds (Ahiflower© oil, Nature’s Crops International, Kensington, Canada) known as the best plant available source of SDA in the nature. Nanno was acquired from Monzón BIOTECH, S.L. (Barcelona, Spain) and Schy was cultivated by Instituto Português do Mar e da Atmosfera (IPMA, Lisboa, Portugal). FA profile of MilkFat, LSO, Buglos, FO, Nanno and Schy diets are shown in Table 1.

### 2.2. Animals and Sample Collection

Animal facilities and environmental requirements, euthanasia and samples collection were previously described [14,19]. Briefly, twenty-four Wistar male rats purchased from Harlan Interfauna Iberica SL (Barcelona, Spain), at the age of 28 days were housed individually and exposed to standard cycles of 12 h light followed by 12 h dark at constant temperature (22 ± 1 °C). After arrival, animals were kept during an adaptation period of 1 week, to end up stress and steady all metabolic conditions. Then, rats were assigned to six body weight-matched groups with four animals each: MilkFat, LSO, Buglos, FO, Nanno and Schy with different dietary n-3 PUFA compositions from vegetable or marine oils. Body weight and feed intake were recorded two times a week. The health status of animals was monitored along the experiment and no casualties were registered. By the end of 10 weeks of experimental trial, rats were fasted for 12 h and killed by decapitation, under light isoflurane (Abbott, IL, USA) anaesthesia. The trunk blood was collected into lithium heparin tubes (Sarstedt, Nümbrecht, Germany) and plasma obtained after centrifugation at 1500× *g* for 10 min. Plasma, liver, brain and visceral white adipose tissue (VAT) from retroperitoneal fat depot were removed, weighed and stored at −80 °C for measurement of fatty acids, and measurement of AEA endocannabinoid and related compounds (NAE).

### 2.3. Lipid Analysis

#### 2.3.1. Measurement of Fatty Acids

Total lipids were extracted from tissues samples (plasma, liver, brain and VAT) according to the method of Folch et al. [33]. Total lipid quantification was performed by the method of Chiang et al. [34]. Aliquots of the lipid fraction were mildly saponified using a procedure in order to obtain unsaturated FA (UFA) for HPLC analysis [35]. All reagents were HPLC grade and purchased from Sigma Chemicals Co. (St. Louis, MO, USA). The separation of FA was carried out using an Agilent 1100 HPLC system (Agilent, Palo Alto, CA, USA) equipped with a diode array detector (DAD). A C-18 Inertsil 5 ODS-2 Chrompack column (Chrompack International BV, Middleburg, The Netherlands) with 5 μm particle size and 150 × 4.6 mm, was used with a mobile phase of CH_3_CN/H_2_O/CH_3_COOH (70/30/0.12, *v*/*v*/*v*) at a flow rate of 1.5 mL/min [36]. Saturated FA (SAFA) were measured as fatty acid methyl esters (FAMEs), by a gas chromatograph (Agilent, Model 6890, Palo Alto) equipped with a flame ionization detector (FID); the split ratio was set at 20:1; the injection port temperature was 270 °C; an autosampler from Agilent (Model 7673, Palo Alto, CA, USA) and a 100 m HP-88 fused capillary column (Agilent, Palo Alto) were used. Data were acquired by the Agilent ChemStation software system.

#### 2.3.2. Measurement of N-acylethanolamines (NAE)

Aliquots of the lipid fraction were used for quantification of NAE compounds. Deuterated NAE and congeners were added as internal standards to the samples before extraction, for quantification by isotope dilution. Internal deuterated standards: [^2^H]_8_AEA, [^2^H]_2_OEA, [^2^H]_4_PEA, [^2^H]_3_SEA were purchased from Cayman Chemicals (MI, USA). NAE quantification was carried out by an Agilent 1100 HPLC system (Agilent, Palo Alto) equipped with a mass spectrometry (MS) Agilent Technologies QQQ triple quadrupole 6420 with electrospray ionization (ESI) source, using positive mode (ESI+). A C-18 Zorbax Eclipse Plus column (Agilent, Palo Alto) with 5 μm particle size and 50 × 4.6 mm was used with a mobile phase of CH_3_OH/H_2_O/CHOOH (80/20/0.1, *v*/*v*/*v*) at a flow rate of 0.5 mL/min.

N_2_ was used as a nebulizing gas with a pressure of 50 psi, drying gas at 300 °C and a flow of 11 L/min, and 4000 V capillary voltage. For each standard, the precursor ion [M+H]^+^ was determined during a full scan (SCAN) in MS and subsequently the obtained product ion (PI) was monitored for each transition in multiple reaction monitoring (MRM) mode in MS/MS. The parameters of source, such as cone voltage or fragmentor (CV) and collision energy (CE) have been optimized for each MRM transition.

Data were acquired by the MassHunter workstation acquisition software (version B.08.02), analyzed with MassHunter software for qualitative (version B.08.00 SP1) and quantitative analyses (version B.09.00). NAE compounds were expressed as mol% of the sum of total FA measured in the corresponding tissue.

### 2.4. Statistical Analysis

The data are expressed as the mean ± SEM of moles of each FA and NAE with respect to total FA (mol%), as specified in the legends.

FA and NAE data were not normally distributed, therefore the differences between the six groups were assessed using nonparametric Kruskal–Wallis test (one-way ANOVA on ranks) followed by Dunn’s correction for multiple comparisons. Correlation studies between each NAE and the respective precursor, and between NAE levels in plasma and their concentration in the different tissues, were done using the Spearman correlation coefficient. 

Data were analyzed using GraphPad Prism 6.0 (GraphPad Software Inc., La Jolla, CA, USA) with *p* ≤ 0.05 as the cut-off for statistical significance between groups. Data with different superscript letters were significantly different according to the statistical analysis, as specified in the tables, and the statistical significances were indicated: * *p* ≤ 0.05; ** *p* ≤ 0.01; *** *p* ≤ 0.001; **** *p* ≤ 0.0001, as specified in the figures.

## 3. Results

After 10 weeks of dietary treatment, the parameters associated with rats’ growth and amount of food intake did not differ among the five groups containing different concentrations and/or composition of n-3 PUFA compared to MilkFat diet (data not shown).

### 3.1. Modification of FA Profiles by n-3 PUFA Diets in Different Tissues

Table 2, Table 3, Table 4 and Table 5 show total FA profiles in several tissues (plasma, liver, brain and VAT) after feeding rats with different dietary n-3 PUFA sources. Tissue FA profiles were strongly influenced by the different dietary FA composition. In fact, in plasma (Table 2) were detected the highest ALA values in LSO- and Buglos-fed animals; EPA in FO- and Nanno-diets; DHA reached similar levels in FO- and Schy-fed animals (34% and 32%, respectively), despite that DHA intake was 3-times higher in Schy relative to the FO diet. Moreover, DHA, the putative metabolite of ALA, did not increase using LSO or Buglos diets. Furthermore, there was a higher conversion of ALA or ALA+SDA to EPA and docosapentanoic acid (DPA, 22:5n-3) in LSO and Buglos groups, and an elevated incorporation of EPA into tissues and the bioconversion of EPA to DPA in the Nanno group was detected. Simultaneously, a significant decrease in n-6 PUFA levels was observed in FO and Schy groups as compared to MilkFat, and there were significant lower AA levels (about 46% of the total FA in FO group). The AA biosynthesis did not change in the Schy or Nanno groups, but in Schy LA (the AA precursor) significantly decreased while eicosatrienoic acid (ETA, 20:3n-6), which is the metabolic AA precursor, presented a trend towards increase as compared to MilkFat.

In the liver (Table 3) the FA profile reflected the one found in plasma with a higher incorporation of total n-3 PUFA with all diets and lower total n-6 PUFA levels compared to MilkFat. No significant changes of AA levels where detected when comparing rats fed MilkFat with all groups, however there was a trend of reduction in FO group (−30%) and of increase in Nanno and Schy groups (+23% and +46% respectively).

In the brain (Table 4) the FA profile slightly changed by using different dietary n-3 PUFA. ALA levels were not different in ALA- and ALA+SDA-enriched LSO and Buglos groups. Moreover, DHA percentage remained unchanged despite its elevated intake using the FO and Schy diets. We found EPA and DPAn-3 increased in all dietary treatments and only EPA significantly in FO group, simultaneously, we found significantly decreased levels of AA and other n-6 PUFA metabolites in the FO-diet group, especially of 22:4n-6 and DPAn-6.

Concerning VAT (Table 5), a significant increase of total n-3 PUFA was detected in LSO group simultaneously to a significant reduction of total n-6 PUFA in LSO and FO groups. In addition, DHA increased significantly in FO and Schy groups.

### 3.2. Effect of n-3 PUFA Diets on the Levels of NAE in Different Tissues

**Figure 1:**
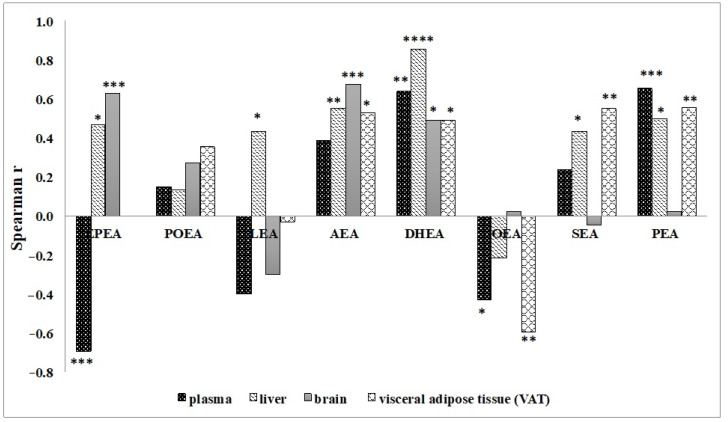
Correlations between NAE species and their respective FA precursors, in plasma, liver, adipose tissue and brain analyzed in Wistar rats fed with different diets. The correlations studies were determined by Spearman correlation coefficient. Statistical significance as follow: * *p* ≤ 0.05; ** *p* ≤ 0.01; *** *p* ≤ 0.001; **** *p* ≤ 0.0001. EPEA, N-eicosapentaenoylethanolamide; POEA, N-pamitoleoylethanolamide; LEA, N-linoleoylethanolamide; AEA, N-arachidonoylethanolamide or anandamide; DHEA, N-docosahexaenoylethanolamide; OEA, N-oleoylethanolamide; SEA, N-stearoylethanolamide; PEA, N-palmitoylethanolamine.

The influence of n-3 PUFA intake with different dietary treatments on the biosynthesis of the main NAE bioactive lipid mediators in relevant tissues of Wistar male rats is detailed in Table 6. Our data indicate that the variations of NAE levels were strongly influenced by tissue FA modifications induced by different dietary n-3 PUFA. A significant positive correlation for nearly all the NAE species with their respective FA precursors in almost all tissues was noted, as described in Figure 1. However, some of them did not follow this trend, such as EPEA in plasma, which had a significant negative correlation with its EPA precursor; also, the LA-derived N-linoleoylethanolamide (LEA) presented a negative, not significant, correlation in plasma, brain and VAT. The oleic acid (OA, 18:1n-9)-derived N-oleoylethanolamide (OEA), showed a significant inverse correlation with OA in plasma and VAT.

In detail, from data reported in Table 6, the levels of AEA did not change in plasma, liver and VAT, though in the latter tissue, the Schy fed group showed a trend of 42% increase when compared to MilkFat. However, in the brain from LSO and FO dietary treatments, the AEA biosynthesis was significantly reduced.

The EPEA levels in plasma decreased significantly only with LSO diet, while in the brain increased significantly in the FO group. The DHEA levels were unchanged in all dietary groups with respect to MilkFat diet. 

**Figure 2:**
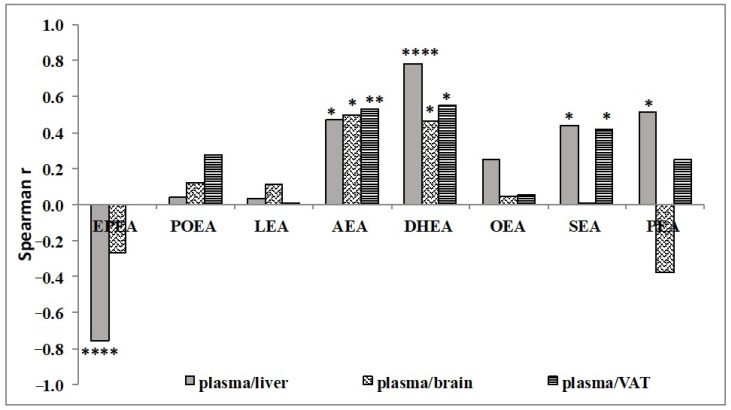
Correlation between levels of NAE species in liver, adipose tissue and brain versus those in plasma in Wistar rats fed with different diets. The correlations studies were determined by Spearman correlation coefficient. Statistical significance as follow: * *p* ≤ 0.05; ** *p* ≤ 0.01; **** *p* ≤ 0.0001. EPEA, N-eicosapentaenoylethanolamide; POEA, N-pamitoleoylethanolamide; LEA, N-linoleoylethanolamide; AEA, N-arachidonoylethanolamide or anandamide; DHEA, N-docosahexaenoylethanolamide; OEA, N-oleoylethanolamide; SEA, N-stearoylethanolamide; PEA, N-palmitoylethanolamine.; VAT, visceral adipose tissue.

The plasma NAE levels correlation with those in tissues may partly reveal the contribution of each tissue on circulating NAE levels (Figure 2). The plasma levels of EPEA presented a significant negative correlation with liver and, not significantly, with brain and therefore it appeared not to derive from liver or brain tissues, while plasmatic AEA and DHEA levels were significantly positively correlated with all the tissues. Moreover, circulating levels of SEA and PEA were positively correlated with liver levels, and SEA also with brain levels.

## 4. Discussion

Our data clearly demonstrate that dietary n-3 PUFA from distinct sources induced differential tissue FA profiles and, consequently, NAE concentrations.

Dietary ALA provided either by LSO or Buglos diets was unable to increase DHA incorporation in any of the tissues analyzed. Interestingly, the inhibition of Δ6-desaturase by ALA from LSO, but not from Buglos, induced a significant decrease of 16% AA levels in brain with respect to the control diet, suggesting that a difference of 27% of dietary ALA may significantly influence AA levels, while the decrease of AA by dietary FO may be attributed to a competition with EPA and DHA for incorporation into phospholipids [8]. These effects on FA modulation appear to be tissue-selective [37,38,39,40,41,42], and our data confirm that the liver is very sensitive to dietary FA modulation with respect to other tissues [43], especially to brain [44]. Nevertheless, only FO showed a significant increase of n-3 HUFA score in all tissues, underlining the synergistic efficacy of dietary EPA and DHA in modulating tissue fatty acid metabolism with important physiological implications [45].

n-6 and n-3 PUFA, besides being essential components of membrane phospholipids, are precursors of bioactive lipid mediators, such as NAE, with specific receptor-mediated physiological activities. Interestingly, it is now well accepted that dietary modifications of the n-6/n-3 PUFA ratio could affect the biosynthesis of NAE, which may be involved in the modulation of metabolic disorders, as neuroinflammation, pro-inflammatory cytokines release, synaptic plasticity and neurodegenerative conditions [46,47,48].

As previously demonstrated in studies with rodents and humans [26,49,50], the present data may suggest an enzymatic competition of the biosynthesis of the various NAE species. Indeed, by modifying the relative proportion of n-3 HUFA in the diet and reducing the n-6/n-3 balance of membrane phospholipids, AEA is expected to decrease. In the brain, different dietary n-3 PUFA, as in LSO and FO, significantly reduced AEA concentrations, while in the liver only FO exerted this effect. The reduction of AEA levels might attenuate its effects on the CB1 cannabinoid receptors, the main molecular target of the endogenous partial agonist AEA, which regulate physiological processes in both the central nervous system and peripheral tissues [51,52,53]. Therefore, in those pathophysiological conditions where is desirable to downregulate an overactive endocannabinoid system, a mixture of EPA and DHA as in FO, and ALA as in LSO, may potentially be a preferred nutritional source of n-3 PUFA. In this regard, it would be interesting to evaluate possible positive metabolic outcomes of a dietary mixture of ALA, EPA and DHA.

In this study, we further confirm that tissue concentrations of some NAE species are positively correlated to their precursors. In particular, this is true for NAE species, such as AEA, DHEA, N-palmitoylethanolamine (PEA) and N-stearoylethanolamine (SEA), derived from FA mainly incorporated into phospholipids, since the NAE FA residues derive from those esterified in the sn-1 position of membrane phospholipids [54]. The brain has been found to be less responsive to dietary FA manipulations probably because it is more resistant to changes in the FA profile [44]. On the other hand, in plasma the correlation cannot be ascribed to a direct relationship precursor-product, but most probably by a similar tissue release of the FA precursor and relative NAE. For example, EPEA was negatively correlated to its plasma precursor, contrarily to liver and brain, indicating that only EPA, but not EPEA, is released from tissues into blood.

This study also approached whether different NAE concentrations in plasma correlate with their own tissue concentrations. This issue is relevant in human studies aimed at evaluating tissue modulation of NAE biosynthesis by dietary FA using circulating levels of NAE as biomarkers. Interestingly, our data showed that changes of AEA and DHEA in the VAT significantly correlated with those in the plasma, suggesting that their systemic levels may reflect changes in the adipose tissue [55]. Interestingly, DHEA plasma levels were also correlated to those in liver and brain, while circulating SEA and PEA may significantly reflect their changes only in the liver. Noteworthy, circulating DHEA is the more changeable NAE species across different tissues. However, more specific studies using tracers should be carried out to confirm the reliability on the use of circulating NAE as tissue biomarkers.

In conclusion, our data indicate that dietary n-3 PUFA of both vegetable and marine origins differently influence not only FA incorporation and biosynthesis in different tissues, but also selectively affect NAE biosynthesis. In particular, given the receptor-mediated tissue specific metabolic role of each NAE, a personalized formulation of dietary n-3 PUFA might potentially produce tailored metabolic effects in different pathophysiological conditions. In addition, since NAE plasma levels may reflect their concentration in specific tissues, it might be feasible to employ circulating NAE levels for evaluating the nutritional impact of n-3 PUFA in humans.

## Figures and Tables

**Table 1:** Fatty acid (FA) profile (%) in different diets.

FA	MilkFat	LSO	Buglos	FO	Nanno	Schy
8:0	2.7	1.87	3.24	1.94	2.56	3.16
10:0	5.99	6.37	8.06	4.42	4.44	5.02
12:0	2.34	3.5	3.69	2.17	2.79	1.23
14:0	8.6	7.24	8.75	9.05	8.07	8.84
15:0	18:57	0.74	0.75	ND	ND	2.16
16:0	27.43	25.29	25.00	27.82	25.94	29.12
16:1n-7	0.99	0.92	0.93	2.41	4.69	1.13
17:0	ND	0.38	ND	0.37	0.34	0.66
18:0	4.43	4.31	3.55	5.58	7.34	5.19
18:1n-9	20.39	20.87	17.7	21.82	19.35	19.43
18:1n-7	0.55	0.88	0.41	0.98	0.44	0.68
18:2n-6	21.33	15.91	14.01	13.79	16.48	16.06
18:3n-6	ND	ND	1.54	ND	ND	ND
18:3n-3	2.61	10.78	7.82	1.72	1.88	1.94
18:4n-3	0.26	0.14	2.53	0.33	0.25	ND
20:4n-6	ND	ND	ND	ND	0.75	ND
20:5n-3	ND	ND	ND	1.4	4.45	ND
22:1n-11	ND	ND	ND	0.98	ND	ND
22:6n-3	ND	ND	ND	1.49	ND	4.08
Others	1.59	0.86	2.02	1.88	0.2	1.34
Oil %	17.92	17.67	17.02	17.47	16.26	16.76
SUM	98.41	99.2	97.98	98.12	99.85	98.70

ND, not detectable.

**Table 2:** Changes of fatty acid (FA) profile in plasma of Wistar rats fed with different diets.

FA	MilkFat	LSO	Buglos	FO	Nanno	Schy
ALA, 18:3n-3	1.5 ± 0.3 ^a,b^	4.8 ± 0.1 ^a^	3.0 ± 0.4 ^a^	0.9 ± 0.0 ^a,b^	0.9 ± 0.1 ^a,b^	0.7 ± 0.1 ^b^
SDA, 18:4n-3	0.1 ± 0.0 ^a^	0.1 ± 0.0 ^a^	0.3 ± 0.0 ^a^	0.1 ± 0.0 ^a^	ND	ND
EPA, 20:5n-3	0.4 ± 0.1 ^b^	1.8 ± 0.1 ^a,b^	1.5 ± 0.1 ^a,b^	3.7 ± 0.1 ^a^	3.9 ± 0.2 ^a^	0.9 ± 0.1 ^a,b^
DPA, 22:5n-3	ND	1.5 ± 0.2 ^a^	1.0 ± 0.2 ^a^	1.6 ± 0.3 ^a^	1.5 ± 0.2 ^a^	ND
DHA, 22:6n-3	1.0 ± 0.1 ^a,b^	1.2 ± 0.1 ^a,b^	0.9 ± 0.1 ^a,b^	3.6 ± 0.1 ^a^	0.6 ± 0.0 ^b^	3.4 ± 0.2 ^a^
LA, 18:2n-6	23.2 ± 0.8 ^a^	21.7 ± 0.4 ^a,b^	20.1 ± 0.5 ^a,b^	20.6 ± 0.4 ^a,b^	21.5 ± 0.3 ^a,b^	15.2 ± 0.6 ^b^
GLA, 18:3n-6	0.3 ± 0.0 ^a,b^	0.2 ± 0.0 ^a,b^	0.7 ± 0.1 ^a^	0.1 ± 0.0 ^b^	0.2 ± 0.0 ^a,b^	0.1 ± 0.0 ^b^
ETA, 20:3n-6	1.1 ± 0.2 ^a,b^	0.8 ± 0.1 ^b^	1.9 ± 0.2 ^a,b^	0.9 ± 0.0 ^a,b^	0.8 ± 0.1 ^a,b^	2.5 ± 0.1 ^a^
AA, 20:4n-6	11.3 ± 0.7 ^a^	8.4 ± 0.4 ^a,b^	8.4 ± 0.2 ^a,b^	6.2 ± 0.3 ^b^	12.1 ± 0.9 ^a^	10.6 ± 0.1 ^a,b^
14:1 n-7	0.2 ± 0.0 ^a^	0.2 ± 0.0 ^a^	0.3 ± 0.0 ^a^	0.4 ± 0.1 ^a^	0.2 ± 0.0 ^a^	0.3 ± 0.0 ^a^
POA, 16:1n-7	1.4 ± 0.2 ^a^	1.6 ± 0.1 ^a^	1.3 ± 0.1 ^a^	2.1 ± 0.2 ^a^	1.8 ± 0.3 ^a^	1.4 ± 0.3 ^a^
OA, 18:1n-9	13.8 ± 1.0 ^a,b^	15.8 ± 0.4 ^a^	14.7 ± 0.3 ^a,b^	15.9 ± 0.2 ^a^	13.0 ± 0.7 ^a,b^	10.9 ±0.6 ^b^
MA, 14:0	2.1 ± 0.3 ^a,b^	0.9 ± 0.3 ^b^	1.4 ± 0.3 ^a,b^	1.8 ± 0.4 ^a,b^	1.8 ± 0.1 ^a,b^	2.8 ± 0.3 ^a^
PA, 16:0	26.8 ± 0.2 ^a,b^	25.3 ± 0.4 ^b^	27.1 ± 0.4 ^a,b^	28.5 ± 0.7 ^a,b^	27.8 ± 0.3 ^a,b^	31.2 ± 0.8 ^a^
SA, 18:0	13.4 ± 0.5 ^a,b^	13.2 ± 0.4 ^a,b^	13.6 ± 0.9 ^a,b^	11.1 ± 0.3 ^b^	11.5 ± 0.3 ^a,b^	14.7 ± 1.0 ^a^
SAFA	42.3 ± 0.5 ^a,b^	39.4 ± 0.4 ^b^	42.1 ± 0.8 ^a,b^	41.5 ± 0.7 ^a,b^	41.1 ± 0.5 ^a,b^	48.7 ± 0.7 ^a^
MUFA	15.4 ± 1.1 ^a,b^	17.7 ± 0.5 ^a,b^	16.3 ± 0.3 ^a,b^	18.4 ± 0.2 ^a^	15.1 ± 0.9 ^a,b^	12.7 ± 0.7 ^b^
PUFA	39.0 ± 1.8 ^a,b^	40.3 ± 1.1 ^a,b^	37.7 ± 1.7 ^a,b^	37.5 ± 0.6 ^a,b^	41.7 ± 0.7 ^a^	33.8 ± 1.2 ^b^
PUFAn-3	2.9 ± 0.4 ^b^	9.0 ± 0.6 ^a^	6.4 ± 0.8 ^a,b^	9.5 ± 0.4 ^a^	6.9 ± 0.4 ^a,b^	5.3 ± 0.4 ^a,b^
PUFAn-6	35.9 ± 0.7 ^a^	31.0 ± 0.6 ^a,b,c^	31.1 ± 0.8 ^a,b^	27.7 ± 0.5 ^b^	34.6 ± 1.0 ^a,c^	28.4 ± 0.8 ^b,c^
n-6/n-3 PUFA	12.8 ± 1.4 ^a^	3.5 ± 0.2 ^b^	5.1 ± 0.7 ^a,b^	2.9 ± 0.2 ^b^	5.0 ± 0.4 ^a,b^	5.4 ± 0.3 ^a,b^
n-3 HUFA score	0.1 ± 0.0 ^a^	0.3 ± 0.0 ^a,b^	0.2 ± 0.0 ^a,b^	0.5 ± 0.0 ^b^	0.3 ± 0.0 ^a,b^	0.3 ± 0.0 ^a,b^

Data represent mean ± SEM for four rats per group and are expressed as % moles/total FA. Values in the same row with different superscript letters are significantly different, *p* ≤ 0.05 (one-way ANOVA nonparametric measures with Kruskal–Wallis test). ND, not detectable. ALA, α-linolenic acid; SDA, stearidonic acid; EPA, eicosapentaenoic acid; DPA, docosapentaenoic acid; DHA, docosahexaenoic acid; LA, linoleic acid; GLA, γ-linolenic acid; ETA, eicosatrienoic acid; AA, arachidonic acid; POA, palmitoleic acid; OA, oleic acid; MA, myristic acid; PA, palmitic acid; SA, stearic acid; SAFA, saturated fatty acids; MUFA, monounsaturated fatty acids; PUFA, polyunsaturated fatty acids; HUFA, high unsaturated fatty acids.

**Table 3:** Changes of fatty acid (FA) profile in liver of Wistar rats fed with different diets.

FA	MilkFat	LSO	Buglos	FO	Nanno	Schy
ALA, 18:3n3	1.7 ±0.1 ^a,b^	8.6 ± 0.2 ^a^	5.5 ± 0.2 ^a^	1.7 ± 0.0 ^a,b^	2.0 ± 0.2 ^a,b^	1.0 ± 0.1 ^b^
SDA, 18:4n-3	0.2 ± 0.0 ^a,b,c^	0.3 ± 0.0 ^a,b^	1.2 ± 0.1 ^a^	0.2 ± 0.0 ^a,b,c^	0.1 ± 0.0 ^b,c^	0.05 ± 0.01 ^c^
EPA, 20:5n-3	0.5 ± 0.0 ^b^	1.7 ± 0.2 ^a,b^	2.0 ± 0.0 ^a,b^	3.7 ± 0.1 ^a^	3.7 ± 0.2 ^a^	1.2 ± 0.2 ^a,b^
DPA, 22:5n-3	1.2 ± 0.3 ^b,c^	2.3 ± 0.1 ^a,b^	1.9 ± 0.1 ^a,b^	2.5 ± 0.1 ^a,c^	3.1 ± 0.1 ^a^	0.8 ± 0.0 ^b^
DHA, 22:6n-3	1.6 ± 0.1 ^b,c^	2.4 ± 0.1 ^a,b,c^	2.2 ± 0.1 ^a,b,c^	7.4 ± 0.1 ^a,b^	1.2 ± 0.0 ^c^	9.0 ± 0.1 ^a^
LA, 18:2n-6	29.9 ± 1.2 ^a^	23.7 ± 0.3 ^a,b^	24.7 ± 0.7 ^a,b^	19.3 ± 0.5 ^b^	22.3 ± 0.7 ^a,b^	20.7 ± 0.6 ^b^
GLA, 18:3n-6	1.0 ± 0.2 ^a,b^	ND	2.1 ± 0.3 ^a^	ND	ND	0.3 ± 0.0 ^b^
ETA, 20:3n-6	1.3 ± 0.1 ^a,b^	1.0 ± 0.1 ^a,b^	1.9 ± 0.2^a^	0.7 ± 0.0 ^b^	1.0 ± 0.1 ^a,b^	3.1 ± 0.1 ^a^
AA, 20:4n-6	6.5 ± 0.1 ^a,b^	5.0 ± 0.6 ^a,b^	5.1 ± 0.1 ^a,b^	4.6 ± 0.1 ^b^	8.0 ± 0.7 ^a^	9.5 ± 0.5 ^a^
DTA, 22:4n-6	0.2 ± 0.0 ^a^	0.1 ± 0.0 ^a,b^	0.1 ± 0.0 ^a,b^	0.2 ± 0.1 ^a,b^	0.1 ± 0.0 ^a,b^	0.1 ± 0.0 ^b^
POA, 16:1n7	3.5 ± 0.5 ^a^	2.6 ± 0.1 ^a^	3.0 ± 0.2 ^a^	4.0 ± 0.3 ^a^	4.7 ± 0.9 ^a^	2.7 ± 0.1 ^a^
OA, 18:1n-9	14.8 ± 0.6 ^a,b^	16.2 ± 0.5 ^a^	14.4 ± 0.6 ^a,b^	17.0 ± 0.6 ^a^	14.6 ± 1.2 ^a,b^	10.9 ± 0.2 ^b^
LAA, 12:0	0.1 ± 0.0 ^a,b^	0.1 ± 0.0 ^a,b^	0.1 ± 0.0 ^a^	0.1 ± 0.0 ^a,b^	0.1 ± 0.0 ^a,b^	0.05 ± 0.0 ^b^
MA, 14:0	1.7 ± 0.1 ^a^	1.8 ± 0.0 ^a^	1.9 ± 0.2 ^a^	2.0 ± 0.1 ^a^	1.7 ± 0.2 ^a^	1.0 ± 0.1 ^a^
PA, 16:0	24.3 ± 0.2 ^a,b^	23.2 ± 0.2 ^b^	24.0 ± 0.5 ^a,b^	26.8 ± 0.6 ^a^	24.9 ± 0.4 ^a,b^	26.4 ± 0.5 ^a^
SA, 18:0	8.5 ± 0.9 ^a,b^	8.0 ± 0.4 ^b^	10.2 ± 0.8 ^a,b^	8.6 ± 0.1 ^a,b^	10.1 ± 1.1 ^a,b^	11.7 ± 0.4 ^a^
SAFA	34.6 ± 0.9 ^a,b^	33.5 ± 0.5 ^b^	36.2 ± 1.1 ^a,b^	37.5 ± 0.8 ^a,b^	36.9 ± 1.3 ^a,b^	39.1 ± 0.4 ^a^
MUFA	18.3 ± 0.9 ^a,b^	18.8 ± 0.5 ^a,b^	17.4 ± 0.7 ^a,b^	21.0 ± 0.7 ^a^	19.3 ± 2.1 ^a,b^	13.7 ± 0.3 ^b^
PUFA	44.3 ± 1.0 ^a,b^	45.5 ± 0.9 ^a,b^	47.2 ± 0.8 ^a^	40.7 ± 0.6 ^b^	42.0 ± 1.1 ^a,b^	45.9 ± 0.4 ^a,b^
PUFAn-3	5.1 ± 0.3 ^b^	15.4 ± 0.2 ^a^	12.9 ± 0.2 ^a,b^	15.5 ± 0.2 ^a^	10.2 ± 0.4 ^a,b^	12.1 ± 0.2 ^a,b^
PUFAn-6	38.9 ± 1.3 ^a^	29.9 ± 0.7 ^a,b^	34.0 ± 0.7 ^a,b^	24.9 ± 0.6 ^b^	31.4 ± 1.3 ^a,b^	33.6 ± 0.3 ^a,b^
n-6/n-3 PUFA	7.7 ± 1.3 ^a^	1.9 ± 0.0 ^b^	2.6 ± 0.1 ^a,b^	1.6 ± 0.1 ^b^	3.1 ± 0.5 ^a,b^	2.8 ± 0.1 ^a,b^
n-3 HUFA score	0.3 ± 0.0 ^b^	0.5 ± 0.0 ^a,b^	0.5 ± 0.0 ^a,b^	0.7 ± 0.0 ^a^	0.5 ± 0.0 ^a,b^	0.5 ± 0.0 ^a,b^

Data represent mean ± SEM for four rats per group and are expressed as % moles/total FA. Values in the same row with different superscript letters are significantly different, *p* ≤ 0.05 (one-way ANOVA nonparametric measures with Kruskal–Wallis test). ND, not detectable. ALA, α-linolenic acid; SDA, stearidonic acid; EPA, eicosapentaenoic acid; DPA, docosapentaenoic acid; DHA, docosahexaenoic acid; LA, linoleic acid; GLA, γ-linolenic acid; ETA, eicosatrienoic acid; AA, arachidonic acid; DTA, docosatetraenoic acid; POA, palmitoleic acid; OA, oleic acid; LAA, lauric acid; MA, myristic acid; PA, palmitic acid; SA, stearic acid; SAFA, saturated fatty acids; MUFA, monounsaturated fatty acids; PUFA, polyunsaturated fatty acids; HUFA, high unsaturated fatty acids.

**Table 4:** Changes of fatty acid (FA) profile in brain of Wistar rats fed with different diets.

FA	MilkFat	LSO	Buglos	FO	Nanno	Schy
ALA, 18:3n3	0.04 ± 0.01 ^a^	0.05 ± 0.01 ^a^	0.06 ± 0.01 ^a^	0.04 ± 0.01 ^a^	0.04 ± 0.00 ^a^	0.04 ± 0.01 ^a^
EPA, 20:5n-3	0.01 ± 0.00 ^b^	0.04 ± 0.00 ^a,b^	0.05 ± 0.00 ^a,b^	0.10 ± 0.01 ^a^	0.05 ± 0.00 ^a,b^	0.04 ± 0.00 ^b^
DPA, 22:5n-3	0.37 ± 0.07 ^a,b^	0.50 ± 0.02 ^a,b^	0.53 ± 0.11 ^a,b^	0.62 ± 0.07 ^a,b^	0.65 ± 0.05 ^a^	0.27 ± 0.04 ^b^
DHA, 22:6n-3	9.16 ± 0.26 ^a,b^	8.52 ± 0.14 ^b^	9.02 ± 0.05 ^a,b^	9.23 ± 0.40 ^a,b^	9.16 ± 0.25 ^a,b^	9.93 ± 0.33 ^a^
LA, 18:2n-6	1.53 ± 0.13 ^a,b^	1.66 ± 0.05 ^a,b^	1.77 ± 0.06 ^a,b^	1.71 ± 0.05 ^a,b^	1.88 ± 0.09 ^a^	1.23 ± 0.04 ^b^
ETA, 20:3n-6	0.36 ± 0.09 ^a,b^	0.39 ± 0.02 ^a,b^	0.62 ± 0.08 ^a^	0.49 ± 0.01 ^a,b^	0.31 ± 0.01 ^b^	0.34 ± 0.03 ^a,b^
AA, 20:4n-6	9.55 ± 0.24 ^a^	8.06 ± 0.07 ^b^	8.99 ± 0.17 ^a,b^	7.88 ± 0.17 ^b^	8.88 ± 0.06 ^a,b^	8.43 ± 0.12 ^a,b^
DTA, 22:4n-6	2.07 ± 0.04 ^a,b^	1.79 ± 0.02 ^a,b,c^	2.01 ± 0.03 ^a,b^	1.53 ± 0.02 ^c^	1.88 ± 0.01 ^a,b,c^	1.64 ± 0.07 ^b,c^
DPA, 22:5n-6	0.52 ± 0.09 ^a^	0.21 ± 0.03 ^a,b^	0.24 ± 0.06 ^a,b^	0.14 ± 0.00 ^b^	0.27 ± 0.02 ^a,b^	0.61 ± 0.04 ^a^
POA, 16:1n7	0.78 ± 0.06 ^a^	0.62 ± 0.05 ^a^	0.69 ± 0.04 ^a^	0.83 ± 0.05 ^a^	0.77 ± 0.03 ^a^	0.70 ± 0.02 ^a^
OA, 18:1n-9	19.34 ± 0.84 ^a^	19.88 ± 0.39 ^a^	20.01 ± 0.36 ^a^	19.85 ± 0.81 ^a^	18.88 ± 0.47 ^a^	20.15 ± 0.73 ^a^
LAA, 12:0	0.34 ± 0.03 ^a,b^	0.22 ± 0.02 ^b^	0.29 ± 0.02 ^a,b^	0.27 ± 0.03 ^a,b^	0.33 ± 0.02 ^a,b^	0.42 ± 0.04 ^a^
MA, 14:0	2.21 ± 0.12 ^b^	3.06 ± 0.16 ^a^	2.29 ± 0.14 ^a,b^	2.71 ± 0.16 ^a,b^	2.53 ± 0.08 ^a,b^	2.31 ± 0.13 ^a,b^
PA, 16:0	31.35 ± 0.43 ^a^	31.87 ± 0.14 ^a^	30.91 ± 0.38 ^a^	31.94 ± 0.69 ^a^	31.73 ± 0.13 ^a^	31.23 ± 0.27 ^a^
SA, 18:0	19.57 ± 0.15 ^a^	20.18 ± 0.31 ^a^	19.89 ± 0.06 ^a^	20.14 ± 0.40 ^a^	19.79 ± 0.12 ^a^	19.64 ± 0.1 ^a^
SAFA	53.46 ± 0.52 ^a^	55.33 ± 0.31 ^a^	53.38 ± 0.46 ^a^	55.06 ± 1.12 ^a^	54.38 ± 0.19 ^a^	53.60 ± 0.29 ^a^
MUFA	20.12 ± 0.81 ^a^	20.50 ± 0.37 ^a^	20.70 ± 0.35 ^a^	20.68 ± 0.84 ^a^	19.65 ± 0.48 ^a^	20.85 ± 0.75 ^a^
PUFA	24.00 ± 0.48 ^a^	21.62 ± 0.17 ^b^	23.91 ± 0.16 ^a,b^	22.24 ± 0.68 ^a,b^	23.44 ± 0.48 ^a,b^	22.88 ± 0.36 ^a,b^
PUFAn-3	9.59 ± 0.26 ^a^	9.12 ± 0.14 ^a^	9.65 ± 0.14 ^a^	9.99 ± 0.47 ^a^	9.90 ± 0.30 ^a^	10.28 ± 0.32 ^a^
PUFAn-6	14.39 ± 0.28 ^a^	12.49 ± 0.08 ^a,b^	14.25 ± 0.14 ^a^	12.24 ± 0.22 ^b^	13.53 ± 0.19 ^a,b^	12.59 ± 0.12 ^a,b^
n-6/n-3 PUFA	1.50 ± 0.06 ^a^	1.37 ± 0.04 ^a,b^	1.48 ± 0.06^a^	1.23 ± 0.08 ^b^	1.37 ± 0.05 ^a,b^	1.23 ± 0.08 ^b^
n-3 HUFA score	0.43 ± 0.01 ^b^	0.46 ± 0.01 ^a,b^	0.45 ± 0.01 ^a,b^	0.50 ± 0.02 ^a^	0.46 ± 0.01 ^a,b^	0.48 ± 0.02 ^a^

Data represent mean ± SEM for four rats per group and are expressed as % moles/total FA. Values in the same row with different superscript letters are significantly different, *p* ≤ 0.05 (one-way ANOVA nonparametric measures with Kruskal–Wallis test). ALA, α-linolenic acid; EPA, eicosapentaenoic acid; DHA, docosahexaenoic acid; LA, linoleic acid; ETA, eicosatrienoic acid; AA, arachidonic acid; DTA, docosatetraenoic acid; DPA, docosapentaenoic acid; POA, palmitoleic acid; OA, oleic acid; LAA, lauric acid; MA, myristic acid; PA, palmitic acid; SA, stearic acid; SAFA, saturated fatty acids; MUFA, monounsaturated fatty acids; PUFA, polyunsaturated fatty acids; HUFA, high unsaturated fatty acids.

**Table 5:** Changes of fatty acid (FA) profile in visceral adipose tissue (VAT) of Wistar rats fed with different diets.

FA	MilkFat	LSO	Buglos	FO	Nanno	Schy
ALA, 18:3n3	2.12 ± 0.06 ^a,b^	5.88 ± 0.76 ^a^	5.23 ± 0.80 ^a^	1.23 ± 0.12 ^b^	1.82 ± 0.07 ^a,b^	1.64 ± 0.06 ^a,b^
SDA, 18:4n-3	0.01 ± 0.00 ^a,b^	0.01 ± 0.00 ^a,b^	0.55 ± 0.14 ^a^	0.09 ± 0.01 ^a,b^	0.01 ± 0.00 ^b^	0.01 ± 0.00 ^a,b^
EPA, 20:5n-3	0.02 ± 0.00 ^b^	0.06 ± 0.01 ^a,b^	0.07 ± 0.01 ^a,b^	0.19 ± 0.02 ^a,b^	0.39 ± 0.01 ^a^	0.07 ± 0.01 ^a,b^
DPA, 22:5n-3	0.06 ± 0.01 ^b^	0.12 ± 0.02 ^a,b^	0.06 ± 0.02 ^b^	0.21 ± 0.02 ^a,b^	0.34 ± 0.04 ^a^	0.11 ± 0.02 ^a,b^
DHA, 22:6n-3	0.03 ± 0.00 ^b^	0.05 ± 0.00 ^b^	0.12 ± 0.01 ^a,b^	0.40 ± 0.01 ^a^	0.07 ± 0.01 ^a,b^	1.32 ± 0.08 ^a^
LA, 18:2n-6	23.90 ± 0.17 ^a^	14.81 ± 1.62 ^b^	21.38 ± 1.49 ^a,b^	14.76 ± 0.79 ^b^	20.45 ± 0.83 ^a,b^	19.63 ± 0.78 ^a,b^
GLA, 18:3n-6	0.07 ± 0.01 ^a,b^	0.08 ± 0.01 ^a,b^	0.72 ± 0.18 ^a^	0.05 ± 0.01 ^b^	0.07 ± 0.00 ^a,b^	0.07 ± 0.01 ^a,b^
ETA, 20:3n-6	0.12 ± 0.01 ^a,b^	0.09 ± 0.02 ^b^	0.22 ± 0.02 ^a^	0.12 ± 0.01 ^a,b^	0.15 ± 0.01 ^a,b^	ND
AA, 20:4n-6	0.20 ± 0.02 ^a,b^	0.14 ± 0.02 ^b^	0.18 ± 0.01 ^a,b^	0.15 ± 0.01 ^a,b^	0.29 ± 0.01 ^a^	0.30 ± 0.02 ^a^
14:1n-5	0.25 ± 0.02 ^a^	0.23 ± 0.04 ^a^	0.30 ± 0.03 ^a^	0.26 ± 0.02 ^a^	0.36 ± 0.02 ^a^	0.29 ± 0.02 ^a^
POA, 16:1n7	1.96 ± 0.14 ^b^	1.69 ± 0.27 ^b^	2.52 ± 0.12 ^a,b^	2.91 ± 0.25 ^a,b^	3.82 ± 0.20 ^a^	2.53 ± 0.41 ^a,b^
OA, 18:1n-9	27.83 ± 0.28 ^a^	23.69 ± 3.36 ^a^	32.64 ± 0.79 ^a^	25.47 ± 1.46 ^a^	26.03 ± 0.32 ^a^	24.71 ± 1.60 ^a^
LAA, 12:0	1.70 ± 0.10 ^a^	1.97 ± 0.22 ^a^	1.51 ± 0.05 ^a^	2.06 ± 0.14 ^a^	1.71 ± 0.10 ^a^	1.86 ± 0.15 ^a^
MA, 14:0	5.35 ± 0.15 ^a,b^	6.76 ± 0.75 ^a,b^	4.83 ± 0.23 ^b^	7.87 ± 0.46 ^a^	6.03 ± 0.36 ^a,b^	6.37 ± 0.46 ^a,b^
PA, 16:0	28.16 ± 0.46 ^a,b^	34.73 ± 4.28 ^a,b^	23.92 ± 1.66 ^b^	35.65 ± 1.74 ^a^	28.82 ± 1.65 ^a,b^	31.58 ± 1.80 ^a,b^
SA, 18:0	4.66 ± 0.13 ^a^	5.47 ± 0.47 ^a^	4.00 ± 0.30 ^a^	5.40 ± 0.32 ^a^	4.73 ± 0.49 ^a^	4.97 ± 0.36 ^a^
SAFA	30.42 ± 10.14 ^a,b^	49.46 ± 5.68 ^a,b^	34.83 ± 2.17 ^b^	51.39 ± 2.64 ^a^	41.68 ± 2.49 ^a,b^	45.52 ± 2.75 ^a,b^
MUFA	30.04 ± 0.18 ^a,b^	25.60 ± 3.65 ^a,b^	35.46 ± 0.65 ^a^	28.65 ± 1.71 ^a,b^	30.22 ± 0.44 ^a,b^	27.53 ± 1.81 ^b^
PUFA	26.95 ± 0.30 ^a,b^	21.63 ± 2.46 ^a,b^	29.06 ± 0.80 ^a^	13.20 ± 4.47 ^b^	24.11 ± 0.88 ^a,b^	24.37 ± 0.94 ^a,b^
PUFAn-3	2.24 ± 0.07 ^b,c^	6.11 ± 0.77 ^a^	6.03 ± 0.95 ^a,b^	2.12 ± 0.15 ^c^	2.61 ± 0.03 ^a,b,c^	3.15 ± 0.15 ^a,b,c^
PUFAn-6	24.33 ± 0.20 ^a^	15.14 ± 1.67 ^b^	22.53 ± 1.33 ^a,b^	15.11 ± 0.81 ^b^	21.01 ± 0.84 ^a,b^	20.84 ± 0.94 ^a,b^
n-6/n-3 PUFA	10.87 ± 0.54 ^a^	2.51 ± 0.23 ^b^	4.26 ± 2.30 ^a,b^	7.16 ± 0.58 ^a,b^	8.03 ± 0.55 ^a^	6.67 ± 0.98 ^a,b^
n-3 HUFA score	0.24 ± 0.02 ^b^	0.48 ± 0.08 ^a,b^	0.37 ± 0.04 ^b^	0.73 ± 0.02 ^a^	0.61 ± 0.04 ^a,b^	0.58 ± 0.11 ^a,b^

Data represent mean ± SEM for four rats per group and are expressed as % moles/total FA. Values in the same row with different superscript letters are significantly different, *p* ≤ 0.05 (one-way ANOVA nonparametric measures with Kruskal–Wallis test). ND, not detectable. ALA, α-linolenic acid; SDA, stearidonic acid; EPA, eicosapentaenoic acid; DPA, docosapentaenoic acid; DHA, docosahexaenoic acid; LA, linoleic acid; GLA, γ-linolenic acid; ETA, eicosatrienoic acid; AA, arachidonic acid; POA, palmitoleic acid; OA, oleic acid; LAA, lauric acid; MA, myristic acid; PA, palmitic acid; SA, stearic acid; SAFA, saturated fatty acids; MUFA, monounsaturated fatty acids; PUFA, polyunsaturated fatty acids; HUFA, high unsaturated fatty acids.

**Table 6:** Changes of N-acylethanolamines (NAE) profile in key metabolic tissues of Wistar rats fed with different diets.

NAE	MilkFat	LSO	Buglos	FO	Nanno	Schy
**Plasma**						
EPEA	117.6 ± 11.2 ^a^	19.7 ± 3.5 ^b^	72.7 ± 12.3 ^a,b^	35.5 ± 12.4 ^a,b^	43.5 ± 10.3 ^a,b^	108.0 ± 7.4 ^a^
POEA	11.9 ± 0.7 ^a^	10.7 ± 1.2 ^a^	13.7 ± 2.5 ^a^	14.2 ± 4.8 ^a^	14.6 ± 1.7 ^a^	19.2 ± 0.8 ^a^
LEA	60.7 ± 2.9 ^a^	52.3 ± 4.0 ^a^	70.2 ± 12.2 ^a^	44.2 ± 15.0 ^a^	65.2 ± 1.9 ^a^	67.8 ± 5.2 ^a^
AEA	25.1 ± 1.9 ^a^	17.5 ± 1.5 ^a^	22.8 ± 3.0 ^a^	13.1 ± 4.6 ^a^	22.5 ± 1.3 ^a^	25.9 ± 1.3 ^a^
DHEA	42.3 ± 3.0 ^a,b^	37.3 ± 0.7 ^a,b^	43.3 ± 5.1 ^a,b^	60.0 ± 2.1 ^a,b^	37.6 ± 2.9 ^b^	138.8 ± 7.5 ^a^
OEA	142.0 ± 9.1 ^a^	118.9 ± 14.7 ^a^	140.0 ± 14.0 ^a^	118.7 ± 41.8 ^a^	147.3 ± 4.3 ^a^	165.6 ± 10.5 ^a^
SEA	237.2 ± 26.2 ^a,b^	179.9 ± 14.6 ^a,b^	163.9 ± 16.9 ^b^	193.3 ± 48.8 ^a,b^	224.5 ± 10.1 ^a,b^	319.6 ± 28.6 ^a^
PEA	181.7 ± 13.1 ^a,b^	167.8 ± 27.6 ^b^	220.1 ± 26.7 ^a,b^	213.3 ± 22.0 ^a,b^	193.5 ± 16.7 ^a,b^	275.2 ± 6.8 ^a^
**Liver**						
EPEA	52.4 ± 8.5 ^a,b^	163.6 ± 12.9 ^a^	27.1 ± 6.6 ^b^	161.3 ± 15.3 ^a^	112.2 ± 15.9 ^a,b^	52.4 ± 12.8 ^a,b^
POEA	5.8 ± 1.2 ^a^	7.9 ± 1.6 ^a^	4.4 ± 1.3 ^a^	10.3 ± 1.4 ^a^	4.9 ± 0.7 ^a^	4.6 ± 0.8 ^a^
LEA	188.8 ± 16.7 ^a^	111.4 ± 7.6 ^a,b^	105.6 ± 6.8 ^a,b^	104.0 ± 5.1 ^a,b^	81.3 ± 8.3 ^b^	117.4 ± 5.2 ^a,b^
AEA	51.1 ± 9.0 ^a,b^	34.2 ± 2.5 ^a,b^	34.1 ± 3.9 ^a,b^	24.3 ± 4.3 ^b^	31.5 ± 7.1 ^a,b^	53.8 ± 3.3 ^a^
DHEA	13.2 ± 1.2 ^a,b,c^	13.1 ± 1.3 ^a,b,c^	10.1 ± 0.5 ^b,c^	42.0 ± 5.3 ^a,b^	6.3 ± 1.1 ^c^	83.9 ± 6.3 ^a^
OEA	58.2 ± 13.3 ^a^	56.2 ± 6.1 ^a^	48.7 ± 1.3 ^a^	47.0 ± 4.8 ^a^	32.6 ± 4.9 ^a^	51.2 ± 6.7 ^a^
SEA	32.3 ± 1.1 ^a,b^	49.5 ± 1.7 ^a,b^	54.8 ±5.0 ^a^	50.8 ± 3.3 ^a,b^	22.0 ± 1.8 ^b^	60.1 ± 7.2 ^a^
PEA	33.3 ± 3.9 ^a,b^	26.1 ± 2.2 ^a,b^	26.7 ± 1.2 ^a,b^	31.1 ± 3.1 ^a,b^	21.6 ± 4.0 ^b^	49.9 ± 3.0 ^a^
**Brain**						
EPEA	19.1 ± 2.9 ^b^	30.3 ± 5.7 ^a,b^	34.3 ± 5.6 ^a,b^	62.0 ± 6.8 ^a^	51.7 ± 14.8 ^a,b^	37.2 ± 6.9 ^a,b^
POEA	110.8 ± 8.5 ^a^	79.2 ± 4.9 ^a^	95.8 ± 5.3 ^a^	110.8 ± 11.3 ^a^	67.7 ± 11.0 ^a^	88.4 ± 7.0 ^a^
LEA	313.1 ± 36.3 ^a^	159.6 ± 3.4 ^b^	222.0 ± 23.0 ^a,b^	191.2 ± 19.2 ^a,b^	148.9 ± 31.5 ^b^	236.6 ± 26.7 ^a,b^
AEA	2181.1 ± 173.0 ^a^	1212.9 ± 24.8 ^b^	1702.2 ± 69.0 ^a,b^	1232.6 ± 62.5 ^b^	1232.1 ± 179.1 ^a,b^	1563.1 ± 88.3 ^a,b^
DHEA	453.1 ± 53.2 ^a^	403.6 ± 3.3 ^a^	363.2 ± 41.0 ^a^	437.9 ± 14.0 ^a^	323.3 ± 39.6 ^a^	468.9 ± 34.7 ^a^
OEA	973.9 ± 74.0 ^a^	804.9 ± 21.2 ^a^	809.3 ± 63.9 ^a^	820.4 ± 50.2 ^a^	619.7 ± 111.4 ^a^	918.7 ± 68.8 ^a^
SEA	321.7 ± 52.2 ^a^	141.0 ± 17.9 ^a^	204.7 ± 81.0 ^a^	251.9 ± 79.6 ^a^	188.3 ± 31.2 ^a^	157.0 ± 11.9 ^a^
PEA	684.3 ± 86.3 ^a^	715.6 ± 85.5 ^a^	649.0 ± 67.2 ^a^	684.5 ± 36.1 ^a^	571.0 ± 145.4 ^a^	691.7 ± 65.7 ^a^
**Visceral adipose tissue (VAT)**						
POEA	0.08 ± 0.00 ^a^	0.09 ± 0.01 ^a^	0.08 ± 0.01 ^a^	0.11 ± 0.01 ^a^	0.11 ± 0.01 ^a^	0.10 ± 0.01 ^a^
LEA	1.16 ± 0.10 ^a^	1.02 ± 0.16 ^a^	0.81 ± 0.06 ^a^	0.86 ± 0.09 ^a^	0.85 ± 0.05 ^a^	1.01 ± 0.04 ^a^
AEA	0.21 ± 0.02 ^a,b^	0.18 ± 0.02 ^a,b^	0.15 ± 0.01 ^b^	0.15 ± 0.02 ^b^	0.20 ± 0.01 ^a,b^	0.30 ± 0.02 ^a^
DHEA	0.36 ± 0.05 ^a,b^	0.33 ± 0.04 ^a,b^	0.23 ± 0.04 ^b^	0.60 ± 0.02 ^a,b^	0.22 ± 0.04 ^b^	1.01 ± 0.06 ^a^
OEA	1.48 ± 0.09 ^a^	1.64 ± 0.25 ^a^	1.18 ± 0.04 ^a^	1.40 ± 0.14 ^a^	1.16 ± 0.09 ^a^	1.43 ± 0.08 ^a^
SEA	2.63 ± 0.04 ^a^	3.11 ± 0.57 ^a^	1.65 ± 0.17 ^a^	2.35 ± 0.22 ^a^	2.33 ± 0.04 ^a^	2.72 ± 0.23 ^a^
PEA	1.68 ± 0.05 ^a^	1.79 ± 0.28 ^a^	1.38 ± 0.04 ^a^	1.52 ± 0.07 ^a^	1.41 ± 0.11 ^a^	1.85 ± 0.11 ^a^

Data represent mean ± SEM for four rats per group and are expressed as %moles/total FA, x1000. Values in the same row with different superscript letters are significantly different, *p* ≤ 0.05 (one-way ANOVA nonparametric measures with Kruskal–Wallis test). AEA, N-arachidonoylethanolamide or anandamide; DHEA, N-docosahexaenoylethanolamide; EPEA, N-eicosapentaenoylethanolamide; LEA, N-linoleoylethanolamide; OEA, N-oleoylethanolamide; PEA, N-palmitoylethanolamine; SEA, N-stearoylethanolamide.

## Data Availability

All relevant data are within the manuscript.

## Abbreviations

AA	arachidonic acid
AEA	N-arachidonoylethanolamide or anandamide
ALA	α-linolenic acid
Buglos	Buglossoides arvensis oil
CE	collision energy
CV	cone voltage
DAD	diode array detector
DHA	docosahexaenoic acid
DHEA	N-docosahexaenoylethanolamide
DPA	docosapentanoic acid
DTA	docosatetraenoic acid
EPA	eicosapentaenoic acid
EPEA	N-eicosapentaenoylethanolamide
ESI	electrospray ionization
ETA	eicosatrienoic acid
FA	fatty acids
FAMEs	fatty acid methyl esters
FID	flame ionization detector
FO	fish oil
GLA	γ-linolenic acid
HUFA	high unsaturated fatty acids
LA	linoleic acid
LAA	lauric acid
LEA	N-linoleoylethanolamide
LSO	linseed oil
MA	myristic acid
MilkFat	milk fat diet
MRM	multiple reaction monitoring
MS	mass spectrometry
NAE	N-acylethanolamides
Nanno	Nannochloropsis microalga oil
OA	oleic acid
OEA	N-oleoylethanolamide
PEA	N-palmitoylethanolamine
PI	product ion
PUFA	polyunsaturated fatty acids
SAFA	saturated fatty acids
Schy	Schizochytrium microalga oil
SDA	stearidonic acid
SA	stearic acid
SEA	N-stearoylethanolamide
VAT	visceral adipose tissue.
